# Validation of an LC-MS/MS-based dilute-and-shoot approach for the quantification of > 500 mycotoxins and other secondary metabolites in food crops: challenges and solutions

**DOI:** 10.1007/s00216-020-02489-9

**Published:** 2020-02-20

**Authors:** Michael Sulyok, David Stadler, David Steiner, Rudolf Krska

**Affiliations:** 1 Department of Agrobiotechnology, IFA-Tulln, Institute of Bioanalytics and Agro-Metabolomics, University of Natural Resources and Life Sciences, Vienna (BOKU), Konrad-Lorenz-Str. 20, 3430 Tulln, Austria; 2 FFoQSI – Austrian Competence Centre for Feed and Food Quality, Safety & Innovation, FFoQSI GmbH, Technopark 1C, 3430 Tulln, Austria; 3 Institute for Global Food Security, School of Biological Sciences, Queen’s University Belfast, University Road, Belfast, Northern Ireland BT7 1NN UK

**Keywords:** Mycotoxins, LC-MS/MS, Recovery, Validation, Multi-analyte methods, Matrix effects

## Abstract

This paper describes the validation of an LC-MS/MS-based method for the quantification of > 500 secondary microbial metabolites. Analytical performance parameters have been determined for seven food matrices using seven individual samples per matrix for spiking. Apparent recoveries ranged from 70 to 120% for 53–83% of all investigated analytes (depending on the matrix). This number increased to 84–94% if the recovery of extraction was considered. The comparison of the fraction of analytes for which the precision criterion of RSD ≤ 20% under repeatability conditions (for 7 replicates derived from different individual samples) and intermediate precision conditions (for 7 technical replicates from one sample), respectively, was met (85–97% vs. 93–94%) highlights the contribution of relative matrix effects to the method uncertainty. Statistical testing of apparent recoveries between pairs of matrices exhibited a significant difference for more than half of the analytes, while recoveries of the extraction showed a much better agreement. Apparent recoveries and matrix effects were found to be constant over 2–3 orders of magnitude of analyte concentrations in figs and maize, whereas the LOQs differed less than by a factor of 2 for 90% of the investigated compounds. Based on these findings, this paper discusses the applicability and practicability of current guidelines for multi-analyte method validation. Investigation of (apparent) recoveries near the LOQ seems to be insufficiently relevant to justify the enormous time-effort for manual inspection of the peaks of hundreds of analytes. Instead, more emphasis should be put on the investigation of relative matrix effects in the validation procedure.

**Electronic supplementary material:**

The online version of this article (10.1007/s00216-020-02489-9) contains supplementary material, which is available to authorized users.

**Figure:**
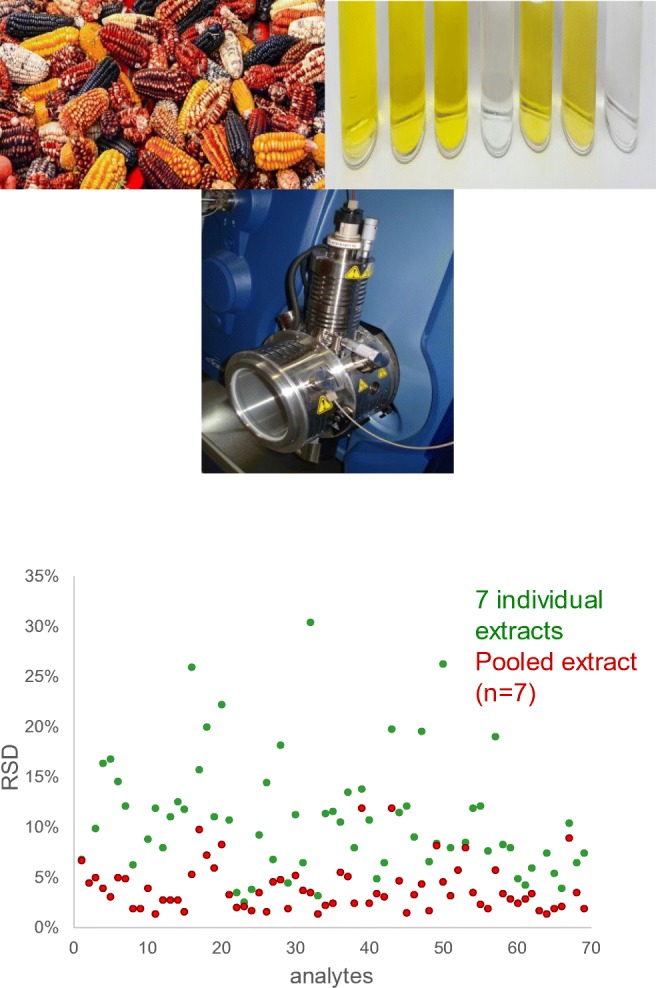
Graphical abstract

## Introduction

Liquid chromatography coupled to tandem mass spectrometry (LC-MS/MS) has become the most intensively used instrumental technique for the quantitative determination of small molecules in food and feed samples. Its high selectivity, sensitivity, robustness, and multi-analyte capability facilitate the simultaneous determination of a large number of analytes from contaminant classes of pesticides, veterinary drugs, mycotoxins, plant toxins, etc., with minimal or even without any clean-up. However, there is a lack of official guidance for the determination of method performance parameters that focus on matrix effects, i.e., the decrease or increase of the analytical response of a given analyte due to co-eluting matrix constituents, which is inherent to LC-MS-based methods for the analysis of diluted crude extracts. This lack of proper and practical guidance documents is particularly true for multi-analyte approaches. It has been emphasized that the workload associated with the validation of such a method requires finding approaches to reduce the analytical burden by, e.g., pooling matrices for validation [1]. Furthermore, it has been stated that it is hardly likely that all compounds of interest in a multi-target method will comply to the criteria that originally have been set for methods tailored for one or few analytes with similar chemical properties [2]. However, an authoritative advice on what compromises are still considered acceptable in view of method performance and workload is missing so far. A revision of performance criteria included in Commission Regulation 401/2006 [3] is currently in progress, which may include description/clarification of “recovery” as meant in the regulation (European Reference Laboratory for mycotoxins and plant toxins, personal communication).

“Recovery” is one term that is insufficiently specified in common (i.e., non-LC-MS specific) guidelines. Part of the existing guidelines uncouple recovery from the measurement process (and thus from the influence of the matrix on the ionization process), e.g., “Proportion of the amount of analyte (…), which is extracted and presented for measurement” [4] or “The proportion of analyte remaining at the final point of determination” [2, 5]. Other guidelines, however, regard recovery in connection with measurement, e.g., “Proportion of analyte determined in the final result compared with the amount added prior to extraction” [6] or “Fraction or percentage of the analyte that is recovered when the test sample is analyzed using the entire method” [7]. IUPAC distinguishes between these two approaches as it has introduced the term “apparent recovery” as an “observed value derived from an analytical procedure by means of a calibration graph divided by a reference value,” which is in contrast to “recovery” that is “the yield of a concentration or extraction step of an analytical process (…)” [8]. Nevertheless, the lack of harmonization in the validation guidelines leaves room for interpretation whether it is “apparent recovery” or “recovery” to which the related performance criteria (e.g., 70–120% in [5]) are to be applied to. As concerns LC-MS-based analysis, the crucial difference is that suppression (or enhancement) of the analytical signal due to co-eluting matrix components contributes to apparent recovery, but not to recovery. This difference may be effectively compensated by stable isotope-labelled internal standards or by matrix-matched calibration, but this may not be feasible for a method covering hundreds of analytes due to the lack of labelled standards (about two dozen isotope-labelled mycotoxins are available) and/or matrix reference materials that are blank for all analytes.

Although signal suppression caused by matrix effects are known to be the Achilles heel of quantitative LC-MS-based analysis, there is very little guidance available on the extent that is considered still acceptable. The criterion given in [5] demands matrix matching if the response in matrix is decreased or increased by more than 20% compared with the solvent standard, but it is not clear whether extreme suppression by, e.g., a factor of 5 or even 10, is still accepted provided it is properly compensated. Indeed, some authors accepted such high matrix effects and still considered their method to be compliant with current regulation, e.g., [9], implying that any target ranges of “recovery” are not to be regarded in connection with matrix effects. In regard to bioanalytical methods, there are differing opinions ranging from a rather strict criterion (tolerance of ± 25% between matrix-matched and external calibration [10]) to a mere statement that a relative response of 100% is not necessary for a reliable bioanalytical assay [11].

However, it might not be absolute but rather relative matrix effects that are the main limitation for the performance of an LC-MS-based multi-method, particularly as they cannot be compensated by matrix-matched calibration. The term “relative matrix” effects has been introduced by Matuszewski et al. [12] who observed significant differences in signal suppression of a drug in different lots of plasma. To take this into account, a maximum RSD of ≤ 15% for matrix effects to be determined in 6 different lots of a biological fluid has been set [11, 13]. A similar numerical value has, to the best of our knowledge, not been set for food analysis. The use of several sources per matrix is recommended in [2], but the related considerations on underestimating the method's uncertainty by using a single matrix source only address “recovery” rather than relative matrix effects in particular. It might be deduced from these recommendations that a method should comply to the criteria set for precision (e.g., RSD_r_ ≤ 20% [5]), even if the data derives from different individual samples. On the other hand, it is recommended to determine repeatability and reproducibility using “identical test items” [14] or “18 aliquots of a blank material” [15]. For this reason, most published methods on LC-MS-based multi-mycotoxin analysis present data on repeatability and reproducibility deriving from a single or a pooled source. This neglects the investigation of relative matrix effects, although it has been observed that their extent might require matching the calibration on a variety level, as it was the case for white and red sorghum [16]. Even without going to such an extreme, we have recently shown that relative matrix effects might significantly contribute to the overall uncertainty of an LC-MS-based method and therefore need to be investigated during validation [17].

A particular challenge in connection with a method covering several hundreds of analytes is the consumption of time for evaluation of raw data. This is especially true for concentrations near the LOQ, which requires manual inspection of the chromatograms, while automatic peak integration is more reliable at levels >> the LOQ. Most guidelines foresee the determination of recoveries at two or three concentration levels [5, 13, 15], as chemisorption might cause very low recoveries at low concentration levels while having no influence at higher levels [18]. Additional samples at low concentrations have to be evaluated to determine LOD and LOQ, e.g., 5 equidistant concentration levels in duplicate, as the use of the signal/noise ratio has been discouraged recently [1]. For the present method, this adds up to 52 samples (3 levels x 7 spiked samples + 3 levels x 7 spiked extracts + 10 samples for LOD/LOQ) x 540 analytes x 2 (quantifier/qualifier) = 56,160 chromatograms per matrix (not taking into consideration external standards and blanks). It is probably for this reason that official guidelines have recognized the unfeasibility of matrix extension validation for each new matrix and have suggested to validate such broad methods for one matrix per matrix group (e.g., matrices with high water, sugar, protein, starch, or fat content [2, 5]) or to pool matrices [1].

In this work, we have for the first time determined the method performance of a dilute-and-shoot method covering more than 500 secondary metabolites including all mycotoxins addressed by regulatory limits as well as emerging and masked mycotoxins in seven different food matrices (wheat, maize, figs, dried grapes, walnuts, pistachios, and almonds). Based on this comprehensive dataset, the applicability and practicability of current guidelines for method validation to such a broad method is discussed. In particular, raw data obtained for apparent recoveries, matrix effects, and recoveries of the extraction step in different matrices, on different concentration levels, and under different conditions (repeatability vs. inter-laboratory reproducibility) have been statistically treated to identify validation experiments which can be skipped in order to significantly reduce the workload required for in-house validation and re-validation upon transfer to a new matrix.

## Materials and methods

### Chemicals and reagents

HiPerSolv Chromanorm HPLC gradient grade acetonitrile was purchased from VWR Chemicals (Vienna, Austria), LC-MS Chromasolv grade methanol was obtained from Honeywell (Seelze, Germany), and LC-MS grade ammonium acetate and glacial acetic acid (p.a.) were purchased from Sigma-Aldrich (Vienna, Austria). A Purelab Ultra system (ELGA LabWater, Celle, Germany) was used for further purification of reverse osmosis water.

Reference standards of mycotoxins and fungal metabolites were either isolated in-house, obtained as gifts, or purchased from the following commercial sources: Romer Labs® Inc. (Tulln, Austria), AnalytiCon Discovery (Potsdam, Germany), Bio Australis (Smithfield, Australia), Fermentek Ltd. (Jerusalem, Israel), AdipoGen Life Sciences (Liestal, Switzerland), BioViotica Naturstoffe GmbH (Dransfeld, Germany), Cfm Oskar Tropitzsch GmbH (Marktredwitz, Germany), Toronto Research Chemicals (Toronto, Canada), Santa Cruz Bioechnology Inc. (Dallas, TE, USA), Sigma-Aldrich (Vienna, Austria), Iris Biotech GmbH (Marktredwitz, Germany), Enzo Life Sciences (Lausen, Switzerland), Chiralix B.V. (Nijmegen, The Netherlands), CSIR Biosciences (Pretoria, South Africa), THP Medical Products (Vienna, Austria), AG Research (Christchurch, New Zealand), Takara Bio Europe (Saint-Germain-En-Laye, France), and LGC Promochem GmbH (Wesel, Germany). The related details are given in Electronic Supplementary Material (ESM) Table S1.

Stock solutions of each analyte were prepared by dissolving the solid substance, preferably at 250 μg/ml in acetonitrile, but depending on the respective solubility, a few compounds were dissolved in acetonitrile/water 1:1 (v/v), methanol or water instead. Sixty-two intermediate mixes were prepared by mixing the stock solutions of 10 analytes each for easier handling. The final multi-analyte standard was freshly prepared prior to spiking experiments by mixing of the intermediate mixes. All solutions were stored at − 20 °C.

### LC-ESI-MS/MS

The method used in this study is an extension of the version described in detail elsewhere [19]. Briefly, a QTrap 5500 MS/MS system (Sciex, Foster City, CA, USA) equipped with a TurboV electrospray ionization (ESI) source was coupled to a 1290 series UHPLC system (Agilent Technologies, Waldbronn, Germany). Chromatographic separation was performed at 25 °C on a Gemini C18-column, 150 × 4.6 mm i.d., 5 μm particle size, equipped with a C18 security guard cartridge, 4 × 3 mm i.d. (both Phenomenex, Torrance, CA, USA). Elution was carried out in binary gradient mode with a flow rate of 1000 μl/min. Both mobile phases contained 5 mM ammonium acetate and were composed of methanol/water/acetic acid 10:89:1 (v/v/v; eluent A) and 97:2:1 (v/v/v; eluent B), respectively. After an initial time of 2 min at 100% A, the proportion of B was increased linearly to 50% within 3 min. Further linear increase of B to 100% within 9 min was followed by a hold time of 4 min at 100% B and 2.5 min column re-equilibration at 100% A. The injection volume was 5 μl. ESI-MS/MS was performed in the scheduled multiple reaction monitoring (sMRM) mode both in positive and negative polarity in two separate chromatographic runs. The target cycle time was 1000 ms, the MS pause time was 3 ms, and the detection window width was 40 and 52 s in the positive and negative ESI mode, respectively. Compound dependent LC-MS/MS parameters are given in ESM Table S2. Two MS/MS transitions were acquired per analyte with the exception of moniliformin and 3-nitroropionic acid that yield only one product ion. For confirmation of a positive identification, the ion ratio has to agree with the related values of the standards within 30% as stated in [20], whereas for the retention time, a more strict in-house criterion of ± 0.03 min is applied.

### Calibration and quantitation

For external neat solvent calibration, a working solution was prepared by mixing 300 μl of the multi-analyte standard with 260 μl of dilution solvent (acetonitrile/water/acetic acid 20/79/1, v/v/v), 20 μl of a certified liquid standard containing fumonisins B1 and B2, and 20 μl of a certified liquid standard containing fumonisin B3 (the fumonisins were added at this late stage as their concentration does not remain stable in the multi-analyte solution of almost pure acetonitrile). From this working solution, a serial dilution was prepared using acetonitrile/water/acetic acid (49.5/49.5/1, v/v/v) to obtain dilution levels of 1:3, 1:10, 1:30, 1:100, 1:300, 1:1000, 1:3000, and 1:1000, respectively. To check the linearity of the response, linear, 1/*x*-weighted calibration curves were constructed for the neat solvent standards. The construction of calibration curves and peak integration was performed using MultiQuant™2.0.2 software (Sciex, Foster City, CA, USA). Further data evaluation, such as the calculation of the method performance parameters, was carried out in Microsoft Excel 2013. *t* tests (two-sided, non-paired, non-equal variance [Welch’s *t* test]) and ANOVA tests ((calculated with one factor (one-way ANOVA)) were performed using R (version 3.1.0 64-bit). For both test variants, a significance level of 0.05 was chosen.

### Spiking of samples and extracts

To 0.25 g of homogenized blank sample, 200 μl of the appropriate spiking solution was added (the working solution was used to obtain spiked samples matched to dilution level 1:10, the external standard on dilution level 1:10 was used to obtain spiked samples matched to dilution level 1:100, etc.). The spiked samples were stored in darkness overnight at room temperature and were subsequently extracted using 1 ml of extraction solvent (acetonitrile/water/acetic acid 79:20:1, v/v/v) and shaken using a rotary shaker (GFL 3017, GFL; Burgwedel, Germany) for 90 min in a horizontal position. The supernatant (300 μl) was transferred into HPLC vials and diluted with the same volume of dilution solvent (acetonitrile/water/acetic acid 20:79:1, v/v/v). After appropriate mixing, 5 μl of the diluted extract was injected into the LC-MS/MS system without further pre-treatment. The spiking protocol was miniaturized to economize on standards; in the routine analysis of naturally contaminated material or materials deriving from proficiency testing, a larger amount of sample (5 or 20 g) is extracted with 20 (or 80) ml of extraction solvent. The related raw extracts are diluted and analyzed as described above.

For post extraction spiking, 500 μl of raw extract was mixed with 100 μl of the appropriate spiking solution and 400 μl of dilution solvent and injected into the LC-MS/MS system as described above.

### Determination of method performance parameters

The recovery of the extraction step (*R*_E_), the apparent recovery (*R*_A_), and the signal suppression/enhancement were calculated from the peak areas of the samples spiked before extraction, the samples spiked after extraction, and the neat solvent standards, respectively, as follows:

$$ {R}_{\mathrm{E}}\ \left(\%\right)=\frac{\mathrm{area}\ \left(\mathrm{sample}\ \mathrm{spiked}\ \mathrm{before}\ \mathrm{extraction}\right)}{\mathrm{area}\ \left(\mathrm{sample}\ \mathrm{spiked}\ \mathrm{after}\ \mathrm{extraction}\right)}\ast 100 $$

$$ {R}_A\ \left(\%\right)=\frac{\mathrm{area}\ \left(\mathrm{sample}\ \mathrm{spiked}\ \mathrm{before}\ \mathrm{extraction}\right)}{\mathrm{area}\ \left(\mathrm{neat}\ \mathrm{solvent}\ \mathrm{standard}\right)}\ast 100 $$

$$ \mathrm{SEE}\ \left(\%\right)=\frac{\mathrm{area}\ \left(\mathrm{sample}\ \mathrm{spiked}\ \mathrm{after}\ \mathrm{extraction}\right)}{\mathrm{area}\ \left(\mathrm{neat}\ \mathrm{solvnt}\ \mathrm{standard}\right)}\ast 100 $$

In the case of wheat, almonds, pistachios, walnuts, and grapes, seven individual samples each were spiked both before and after extraction to match the theoretical concentration with the external standard dilution level 1:10. For maize and figs, the whole procedure of spiking 7 individual samples each before and after extraction was conducted on two additional dilution levels (theoretical concentration matched to the external standards on dilution levels 1:100 and 1:1000) in order to investigate the dependence of *R*_E_, *R*_A_, and SSE on the concentration level.

Finally, spiking of fig and maize samples before extraction was performed on three further levels (theoretical concentration matched to the external standards on dilution levels 1:300, 1:3000, and 1:10000) for the determination of the limit of quantification and the limit of detection according to the EURACHEM guide [21]. The related approach involves replicate measurements of samples with a low concentration of analyte and determination of the standard deviation s_0_ expressed as concentration units. The LOD and LOQ are obtained by multiplying s_0_ with a factor of 3 and 10, respectively.

For the determination of the intermediate precision (within-laboratory reproducibility) in figs, maize, and walnuts, 7 replicates deriving from one individual sample each were spiked on level 1:10 and subsequently analyzed over an extended period (one replicate of each commodity per week) by three different analysts.

### Samples

In order to challenge the repeatability of the extraction protocol and of matrix effects, a heterogeneous set of individual raw samples from field surveys and of processed samples from the supermarket was selected for each matrix (Table 1). Maize and fig samples were described in [17].

**Table 1:** Description of the seven lots of walnuts, pistachios, almonds, raisins, and wheat used as blank samples

	Walnuts	Pistachios	Almonds	Raisins	Wheat
Origin	Afghanistan	Afghanistan	Afghanistan	Afghanistan	Afghanistan
Variety	Korak	Korak	Sattarbai	Tayefee	
Description		Open-shelled, purple skinned	Soft-shelled	Dipped in sulfur	
Origin	Afghanistan	Afghanistan	Afghanistan	Afghanistan	Afghanistan
Variety	Kaghazi	Pushdara	Sattarbai	Gahzni	
Description		Close-shelled, purple skinned	Soft-shelled	Small black-colored seeds	
Origin	Afghanistan	Afghanistan	Afghanistan	Afghanistan	Kazakhstan
Variety	Zard	Khandan-e-Safid	Qambari	Shundurkani	
Description	Yellow kernels	Wrinkly shell	Very strong almond flavor	Golden-colored high value	Sampled in Afghanistan
Origin	Afghanistan	Afghanistan	Afghanistan	Afghanistan	Pakistan
Variety	Mazaari		Sangaki		
Description			Smaller kernels	Long, green, seedless	Sampled in Afghanistan
Origin	Afghanistan	Afghanistan	Afghanistan	Afghanistan	Afghanistan
Variety			Murawaji		
Description			Smaller kernels	Round, green	
Origin	USA/CA	USA	Italy	Iran	Austria
Variety					
Description		Roasted and salted		Contains palm oil	
Origin	Turkey	California	California	Turkey	Austria
Variety					
Description				Contains sun flower oil	

Homogenization was performed using an Osterizer blender (Sunbeam Oster Household Products, Fort Lauderdale, FL, USA). Raisins and figs were immersed in liquid nitrogen prior to blending and the 0.25 g aliquots of ground material used for spiking was taken immediately afterwards.

## Results and discussion

### Method extension

Compared with our previously published method [19], the retention window width in the sMRM mode was reduced while keeping the cycle time of 1 s. The resulting dwell times (dynamically generated from these two parameters and from the number of concurring MRM transitions) were larger than 25 ms with the exception of the period between 9.5 and 10.6 min in the positive ESI mode that exhibited dwell times between 20 and 25 ms. The dwell time issue has been one of the reasons for not switching to UPLC, as it would be necessary to significantly decrease the cycle time (and thus the dwell times) in order to obtain the same number of data points per UPLC peak as for a broader HPLC peak. In addition, robustness of the chromatographic system is an important aspect for a method involving the analysis of crude (and sometimes turbid) extracts. Our LC column has been in use since 8 years without a loss of performance despite an average annual number of 7500 samples and 20,000 injections.

Another minor change is related to citrinin that has been transferred to the negative ESI mode, as the related MRM transitions deriving from the deprotonated methanol adduct [M+CH_3_OH-H]^−^ exhibited a drastically increased signal compared with the positive mode [22].

### Apparent recoveries, matrix effects, and extraction efficiencies

**Fig. 1:**
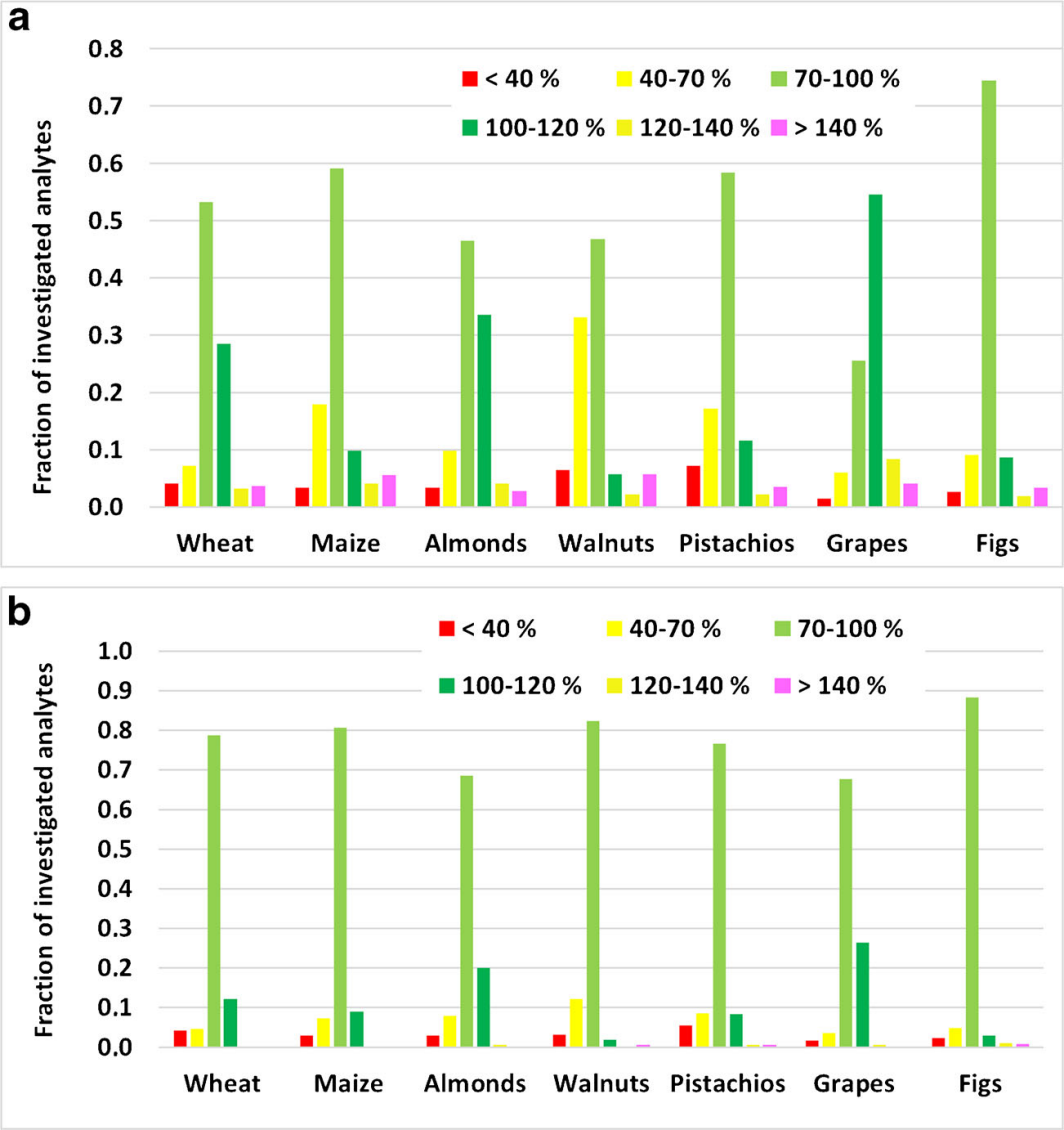
Summary of the apparent recoveries *R*_A_ (**a**) and extraction efficiencies (**b**) obtained for the seven food matrices investigated in this study

All numerical values derived from spiking seven individual samples, each of the seven investigated matrices before and after extraction on dilution level 1:10, are compiled in ESM Table S3. Only 53 (for walnuts) to 83% (for figs) of the apparent recoveries obtained for the different food matrices fell within the target range of 70–120% given in the SANTE guide for pesticides [5] (Fig. 1a). The percentage increases to 84–94% if the recovery of the extraction step rather than the apparent recovery is regarded (Fig. 1b). This discrepancy underlines the need for a harmonized definition of “recovery” (and/or for specification in non-LC-MS-specific guidelines) particularly in view of compliance with the set target ranges. For instance, the apparent recoveries for aflatoxins in maize, walnuts, and pistachios are outside the target range of 80–110% given in the Commission Regulation 401/2006 [3] and Codex Alimentarius [6], whereas the recoveries of the extraction step comply with this criterion for all investigated food matrices.

The reason for the discrepancy between apparent recoveries *R*_A_ and recoveries of the extraction step *R*_E_ is signal suppression or signal enhancement effects (both being referred to as SSE) due to the presence of co-eluting matrix components in the LC-MS/MS interface as described above. Whereas no clear-cut guideline on an acceptable range of matrix effects has been provided for food analysis yet, it has been suggested to classify them as soft (response of matrix-matched standard is ± 20% compared with the solvent standard, i.e., SSE values range from 80 to 100% in case of soft suppression and from 100 to 120% in case of soft enhancement), moderate (from ± 50 to ± 20% compared with the solvent-based standard, i.e., SSE between 50 and 80% in case of medium suppression and 120–150% in case of medium enhancement) and strong (outside ± 50% compared with the solvent-based standard, i.e., SSE < 50% in case of strong suppression and > 150% in case of strong enhancement) [23]. While the effects were soft for > 80% of all investigated analytes in grapes, figs, wheat, and almond, the number of analytes exhibiting medium or strong suppression or enhancement was significantly larger in maize (33%), walnuts (42%), and pistachios (30%) (Table 2).

**Table 2:** Number of analytes attributed to various classes of matrix effects (according to [23])

	Grapes	Figs	Wheat	Maize	Almonds	Walnuts	Pistachios
Strong suppression	1	3	3	2	3	18	8
Medium suppression	17	31	21	91	22	128	89
Soft suppression	81	206	149	251	175	213	225
Soft enhancement	350	234	290	110	283	98	153
Medium enhancement	68	37	51	47	35	41	40
Strong enhancement	19	21	22	37	18	42	26

**Fig. 2:**
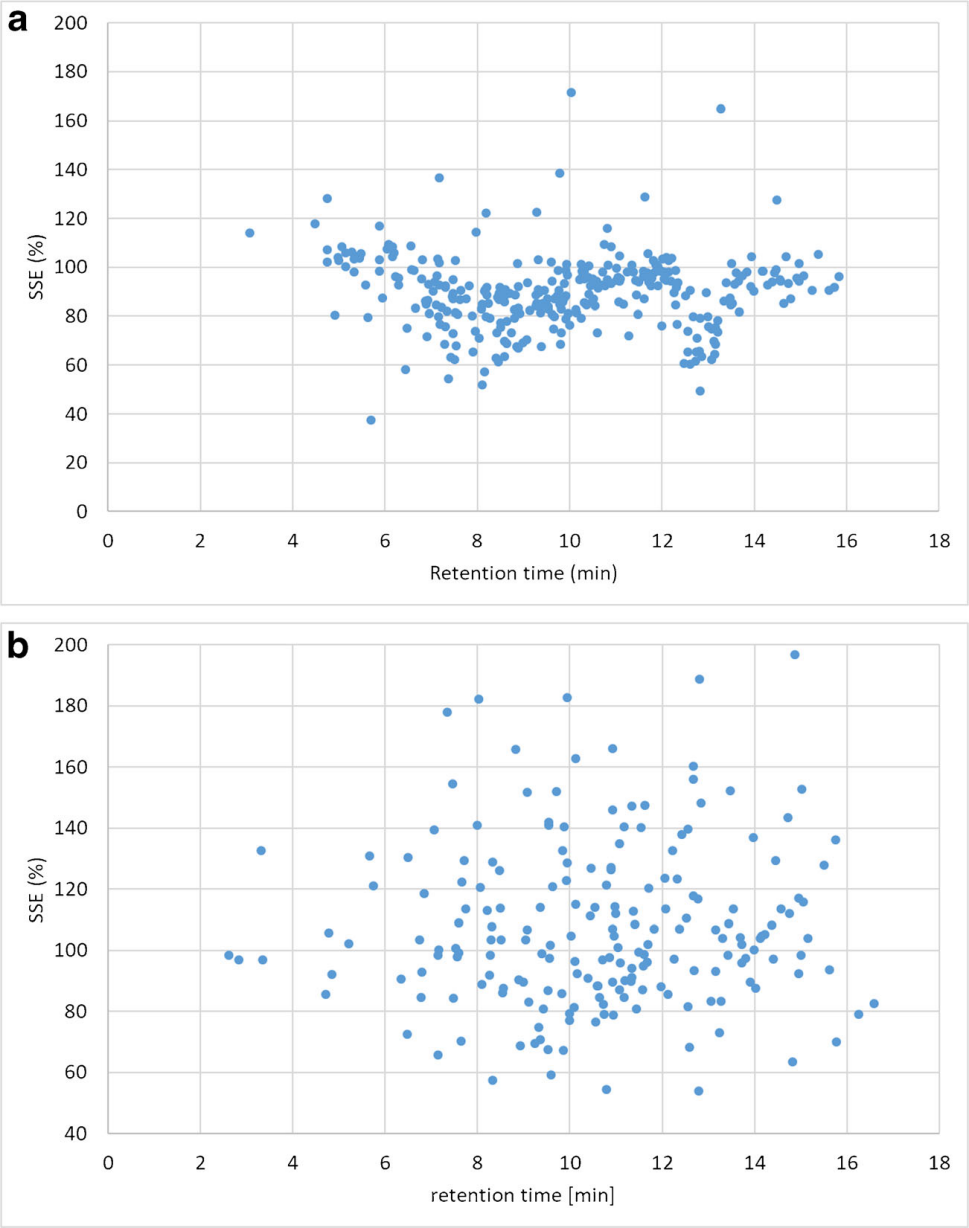
Correlation of matrix effects in maize with retention time. **a**: Positive ionization mode. **b** Negative ionization mode. Each dot represents one analyte

Although matrix effects have been considered to be unpredictable [24] and to depend not only on the matrix but also on the specific analytes, the SSE values were plotted against retention time for all matrices separately for ESI (+) and ESI (−) (Fig. 2) in order to investigate if there is any pattern that would allow a prediction for analytes to be added to the method in the future.

In the positive ionization mode, a rather sharp distribution was observed, with maize exhibiting two retention time segments of generally moderate signal suppression (one between 6 and 10 min and one between 12.5 and 13.2 min). Retention time segments exhibiting a more scattered distribution were observed for walnuts, pistachios, and wheat at rt < 9 min, rt < 8 min, and rt > 14 min, respectively. In the negative ionization mode, the distribution was generally more blurred and there seems to be a tendency towards SSE values > 100% except for walnuts and pistachios.

Consistent strong matrix enhancement has been observed for 15 compounds (asperfuran, antibiotic Y, antibiotic PF 1052, gibberellic acid, demethylsulochrin, tenuazonic acid, barceloneic acid, fumiquinazolin derivative, ochratoxin alpha, atroventinmethylether, viridicatum toxin, equisetin and epi-equisetin, methylequisetin, altersetin). It is unlikely that this phenomenon is a matrix effect in the narrow sense (i.e., related to the ionization process in the ion source); most probably, the matrix acts as protectant against oxygen, light, or active sites in the analytical system that lead to losses of the analyte in the absence of matrix [11].

**Fig. 3:**
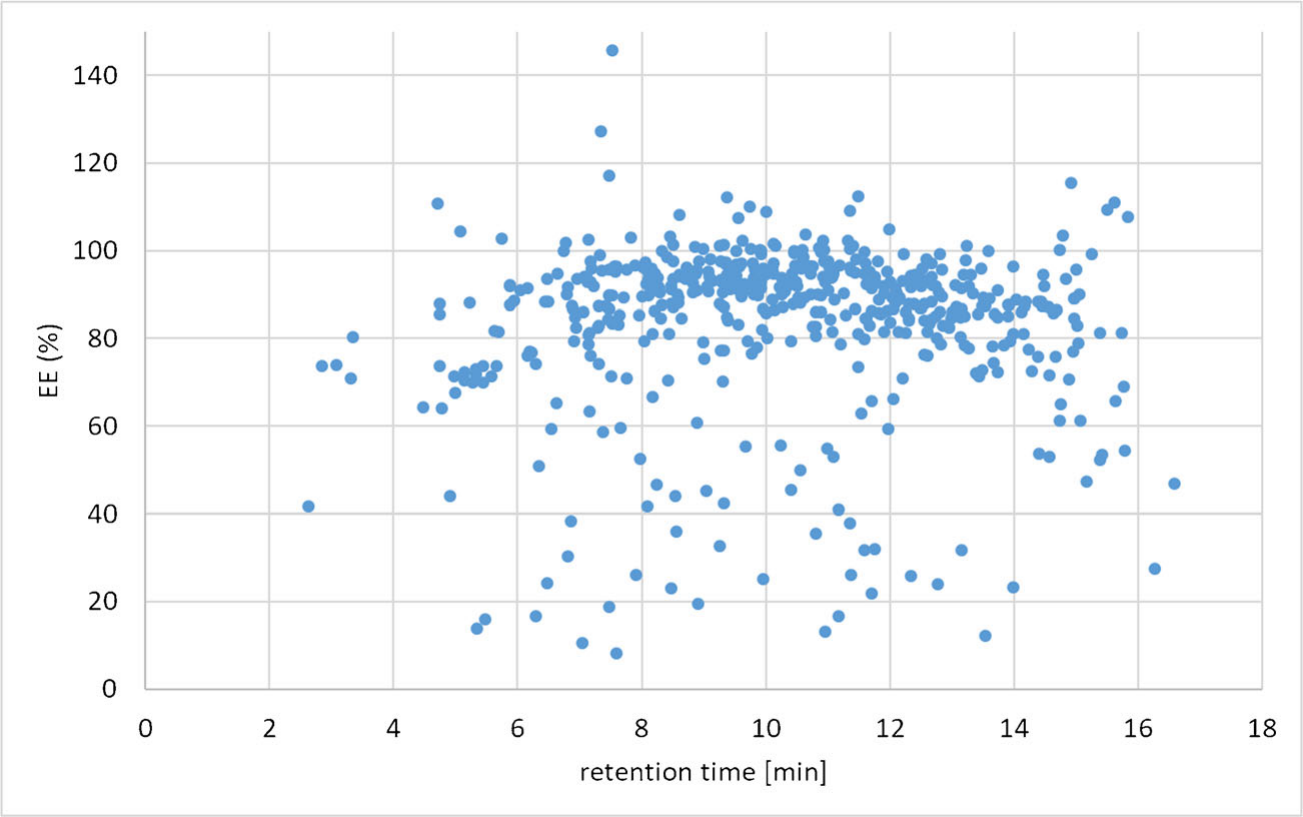
Correlation of recoveries of extraction obtained in pistachios with retention time; each dot represents one analyte

Recoveries of the extraction step have been plotted against retention time as well (see Fig. 3 for pistachios and ESM Fig. S1 for all the other matrices). The majority of the analytes exhibit *R*_E_ values of 80–100% independent from the retention time pointing towards the suitability of the chosen extraction solvent for a wide range of metabolites. In all three nut matrices, the analytes eluting after 13 min exhibited a trend towards somewhat lower *R*_E_ values. Twenty-four analytes exhibited *R*_E_ < 70% in five or more matrices with related RSDs of > 20%, thus indicating a general incompatibility of these substances with the chosen conditions. For 18 compounds (including patulin), the extraction efficiency was compliant in grapes and figs, but not in the nut- or grain-based matrices, whereas nivalenol- and deoxynivalenol glucoside as well as elymoclavin fructoside exhibited particularly low *R*_E_ values in those two matrices.

### Lot-to-lot variation and precision

**Fig. 4:**
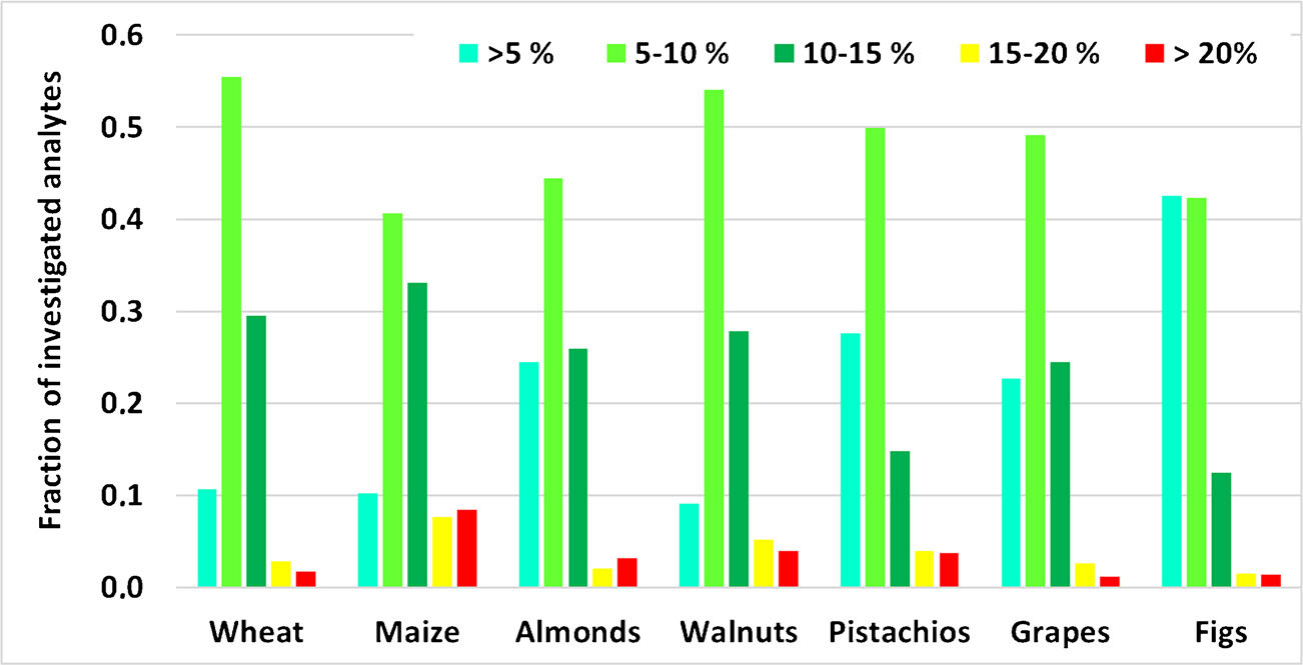
RSDs of signal suppression/enhancement (SSE) obtained for spiked extracts

Considering the criterion of matrix effects having an RSD ≤ 15% in different lots/sources of a given lot in biomedical analysis [10, 11, 13], 84–97% of all results obtained for the seven individual spiked extracts per matrix were compliant (Fig. 4).

**Fig. 5:**
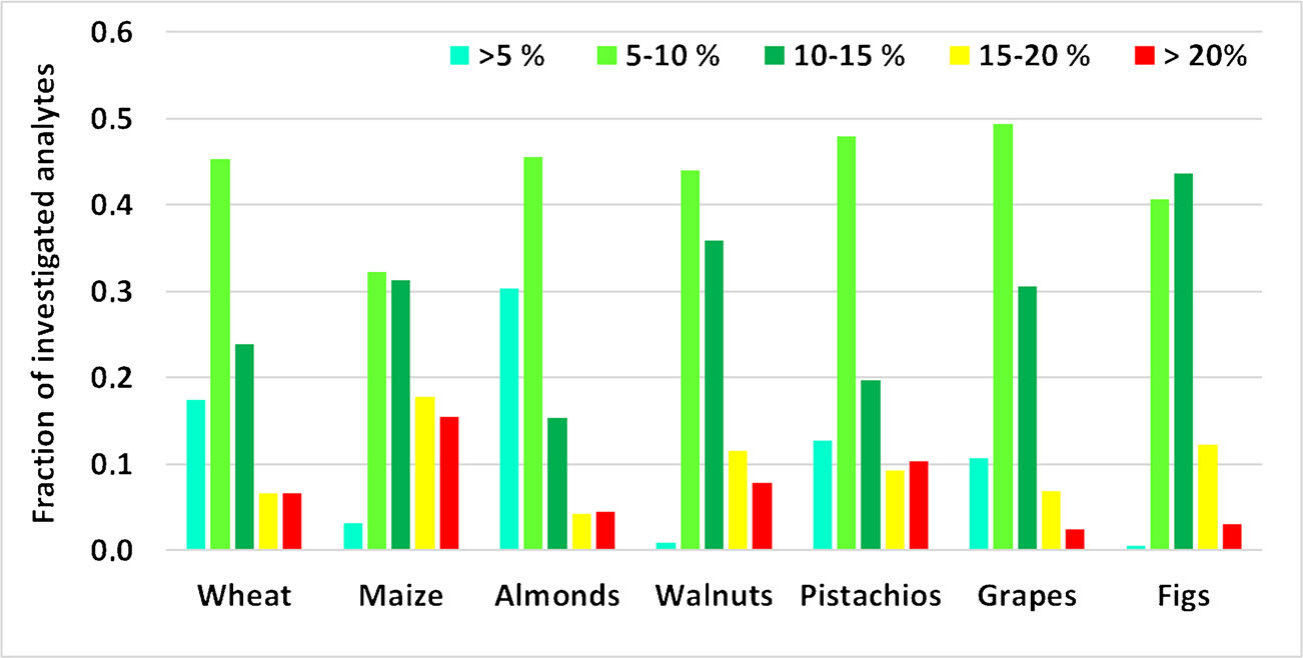
RSD_r_ of apparent recoveries *R*_A_ obtained for spiked samples

Likewise, apparent recoveries of the seven individual samples (per matrix) spiked before extraction under repeatability conditions complied with the RSD ≤ 20% criterion for 85–97% of all analytes (Fig. 5).

The analysis of the variances of spiked samples vs. spiked extracts revealed that there was a significant (*p* < 0.05) difference only for 2–21% of all investigated analytes. This implies that the contribution of the extraction step to the overall variance is only minor compared with that of the relative matrix effects. We have observed that some specific lots behaved somewhat differently in regards matrix effects and/or recovery of the extraction in comparison with the remaining lots. For instance, two maize samples exhibited higher SSE values in the positive ESI mode for some analytes eluting between 8 and 11 min, whereas lower SSE values were obtained for part of the compounds eluting between 12 and 13 min. Likewise, the two pistachio samples purchased from the supermarket exhibited higher extraction efficiencies for some analytes compared with the other five replicates deriving from field sampling. Although these lot-to-lot variations did not result in non-compliance as regards precision for most analyte/matrix combinations, we think that their investigation should be an integral part of any validation process as these variations might significantly contribute to the overall measurement uncertainty of the respective LC-MS/MS method [17]. However, to the best of our knowledge, none of the existing guidelines explicitly demands the spiking of different individual samples for the determination of the lot-to-lot variation. So far, it is common practice to employ replicates derived from the same sample (or from a pooled sample) which are calculated as RSD_r_. Furthermore, lot-to-lot variation should be taken into account by expanding the acceptable RSD_r_ to a value of 25% derived from the currently accepted RSD_r_ ≤ 20% and the RSD ≤ 15% obtained for relative matrix effects due to lot-to-lot variation by following the law of error propagation.

### Intermediate precision (within-laboratory reproducibility)

**Fig. 6:**
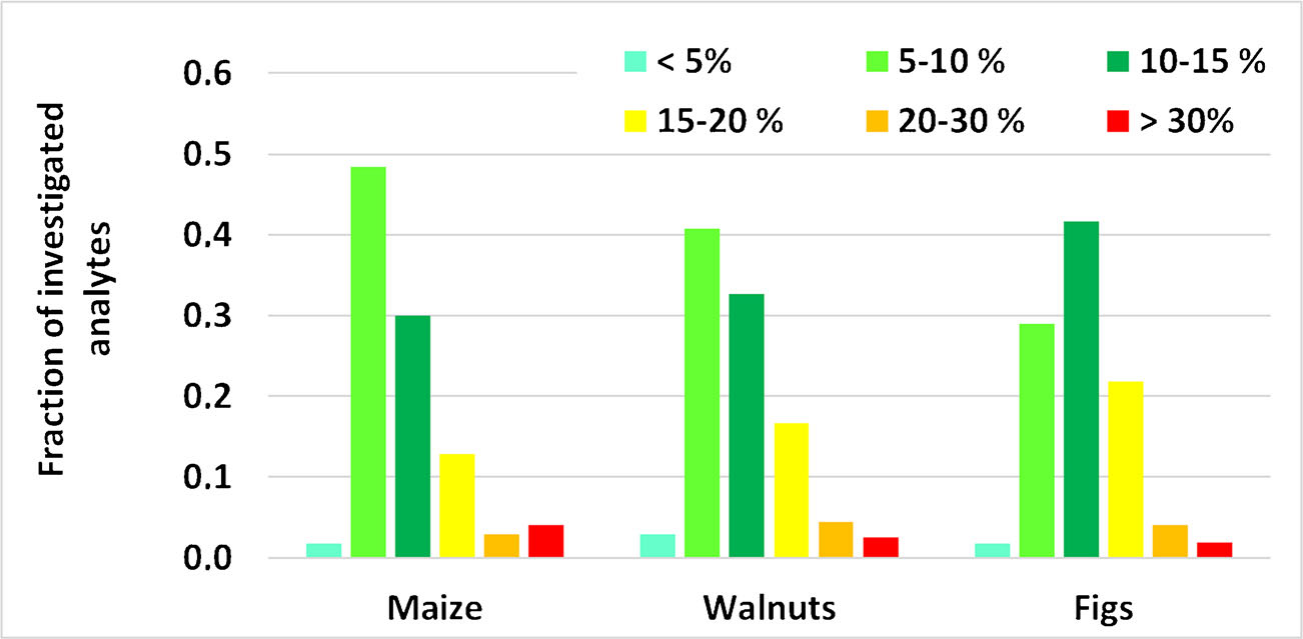
Within-laboratory repeatability obtained for the three investigated matrices

The intermediate precision was determined for maize, walnuts, and figs by spiking one aliquot per matrix derived from only one individual sample each over a period of 7 weeks (performing analysis of one spiked replicate per week for each of the three matrices). The related within-laboratory reproducibility RSD_WLR_ complied with the ≤ 20% criterion set in [5] for 93–94% of all investigated analytes. This fraction increased to 96–98% when the less strict criterion of RSD_WLR_ ≤ 30% from [14] was applied (Fig. 6; all individual values are given in ESM Table S4). These results indicate that both the recovery of the extraction as well as matrix effects remain reasonably constant over time. This is crucial for our approach of performing external instead of matrix-matched calibration, which is due to (1) the non-availability of samples that are true blanks for all analytes and (2) our need to accommodate samples derived from various matrices within one analytical sequence. The fact that the RSD_WLR_ determined from technical replicates of one individual sample is smaller than the RSD_r_ determined from seven individual samples for approx. 60% of all investigated analyte/matrix combinations is another indicator of relative matrix effects being a major contributor to the uncertainty of the method.

### Transferability of apparent recovery and recovery of the extraction step between matrices

Data on apparent recovery and recovery of the extraction obtained for the seven investigated matrices (see ESM Table S3) were *t* tested (*p* < 0.05) for each pair of matrices (see ESM Table S5). Apparent recoveries were significantly different for more than half of the analytes for all pairs except wheat/almond (upper right part of Table 3 (a)), whereas the fraction of significantly different extraction efficiencies was generally in the range between 20 and 40% (lower left part of Table 3 (a)) with a particular good agreement between maize and wheat. If a relative difference of 20% was set as an acceptable criterion for the comparability of the (apparent) recoveries in two matrices, the fraction of non-compliant results was approx. 15–25% when considering extraction efficiency as recovery but remains highly variable (25–75%) when apparent recoveries were considered.

**Table 3:** Comparison of values determined for apparent recovery (italic, upper right part of the table) and extraction efficiency (lower left part). (a) Fraction of analytes (in %) exhibiting significant difference (*p* < 0.05). (b) Fraction of analytes (in %) exhibiting a difference of > 20% rel. Combinations from the same commodity group (grains, nuts, and dried fruits) are given in bold

	Wheat	Maize	Almonds	Walnuts	Pistachios	Grapes	Figs
(a)							
Wheat	**X**	***59.0***	*46.0*	*81.6*	*71.8*	*54.5*	*58.3*
Maize	**9.5**	**X**	*66.6*	*68.8*	*56.3*	*76.1*	*50.0*
Almonds	17.5	23.0	**X**	***83.7***	***70.4***	*55.4*	*62.9*
Walnuts	39.7	49.8	**49.8**	**X**	***62.7***	*85.4*	*70.1*
Pistachios	26.9	35.3	**30.3**	**36.8**	**X**	*80.0*	*53.1*
Grapes	21.5	27.2	22.8	47.3	33.7	**X**	***76.3***
Figs	32.1	38.9	39.7	28.1	33.4	**30.8**	**X**
(b)							
Wheat	**X**	***35.0***	*20.9*	*74.7*	*42.1*	*28.9*	*35.6*
Maize	**8.7**	**X**	*48.2*	*60.0*	*44.8*	*64.9*	*39.4*
Almonds	15.8	15.3	**X**	***68.5***	***45.6***	*26.7*	*26.9*
Walnuts	28.2	23.9	**22.8**	**X**	***47.6***	*79.5*	*57.6*
Pistachios	19.3	21.7	**21.0**	**15.5**	**X**	*56.7*	*34.0*
Grapes	19.9	22.2	20.4	27.9	22.5	**X**	***46.1***
Figs	23.4	16.8	20.2	20.6	20.0	**16.6**	**X**

These results suggest that the transfer of such a broad multi-analyte method to other (similar) matrices without proper re-validation can for some analytes result in serious concessions on the trueness of the measurement results. In particular, the concept of commodity groups is not fully supported by our data, as the agreement in (apparent) recoveries between figs and grapes and within the three nut-based matrices was not significantly better compared with the *R*_A_ results obtained for combinations of different commodity groups.

However, part of our results in proficiency testing (see ESM Table S6) is weakening our previous statement on the need for re-determination of *R*_A_ for every new matrix: These have been submitted for matrices such as cinnamon, paprika, cotton cake, wheat gluten, and crispbread without previously characterizing apparent recoveries for these commodities. Instead, numerical values determined for similar matrices during method validation [19] or during our field studies have been applied. The related *z*-scores do not seem to be significantly worse than those obtained for grain-, nut-, or dried fruit-based matrices more frequently analyzed in our laboratory. Overall, the success rate of *z*-scores between − 2 and 2 is currently > 94%, with the majority of non-compliant results being verified as random errors through re-analysis of the related proficiency test samples. This implies that the systematic errors that are generated through transferring values for *R*_A_ between similar matrices are sufficiently low and thus do not deteriorate the comparability of our method’s results within the offered PT scheme which also considered results based on other detection principles as stated in the related reports. Although we abandoned the use of ^13^C-labelled internal standards, our LC-MS-based method has successfully been applied with a 100% success rate in a related multi-toxin proficiency test for the commodities wheat and maize [25].

### Influence of analyte concentration on method performance data

Data obtained for SSE and *R*_E_ on up to three concentration levels as well as on *R*_A_ on up to six concentration levels, respectively, in both maize and figs has been investigated for statistically significant (*α* = 0.05) differences between the concentration levels. We found that all these parameters remain constant in most cases except for 32 analytes in maize and 29 analytes in figs (see ESM Tables S7 and S8). For SSE, such a concentration independence as observed for aflatoxin B1 in maize (SSE 61 ± 9%, 62 ± 12%, and 62 ± 12% for levels 1:10, 1:100, and 1:1000, respectively) or for fumonisin B3 in figs (SSE 137 ± 3%, 135 ± 8%, and 137 ± 23% for levels 1:10, 1:100, and 1:1000, respectively) was expected (as otherwise the frequently applied concept of matrix matching would result in non-linear calibration functions). However, it is somewhat less expectable that incomplete extraction as observed for citrinin in figs (*R*_E_ 33 ± 2%, 34 ± 3%, and 35 ± 6% for levels 1:10, 1:100, and 1:1000 respectively) and for 3-nitropropioic acid in maize (*R*_E_ 77 ± 8%, 82 ± 6%, and 74 ± 8% for levels 1:10, 1:100, and 1:1000, respectively) remains constant as well. These results underline a major advantage of a dilute-and-shoot approach, i.e., the absence of any clean-up material with a potential population of active sites resulting in particularly low recoveries at low analyte concentration. Considering *R*_A_, the investigation of three additional levels extends the range between the highest and lowest concentration level to 1000 and confirms its independence, e.g., O-methylsterigmatocystin in figs (82 ± 6%, 84 ± 8%, 90 ± 7%, 89 ± 13%, 81 ± 11%, and 89 ± 12% for levels 1:10, 1:100, 1:300, 1:1000, 1:3000, and 1:10000, respectively) or ochratoxin C in maize (58 ± 12%, 61 ± 12%, 56 ± 5%, 67 ± 15%, 58 ± 8%, and 60 ± 12% for levels 1:10, 1:100, 1:300, 1:1000, 1:3000, and 1:10000, respectively). In view of these results, one may question whether it is essential to determine method performance data at low concentration levels for each new matrix. Instead, spiking at one very high level seems to be sufficient which facilitates data evaluation as there is no need for manual inspection of automatic peak integration and for correction of the background concentration in the samples used as “blanks” for spiking.

### Limits of quantification and limits of detection

**Fig. 7:**
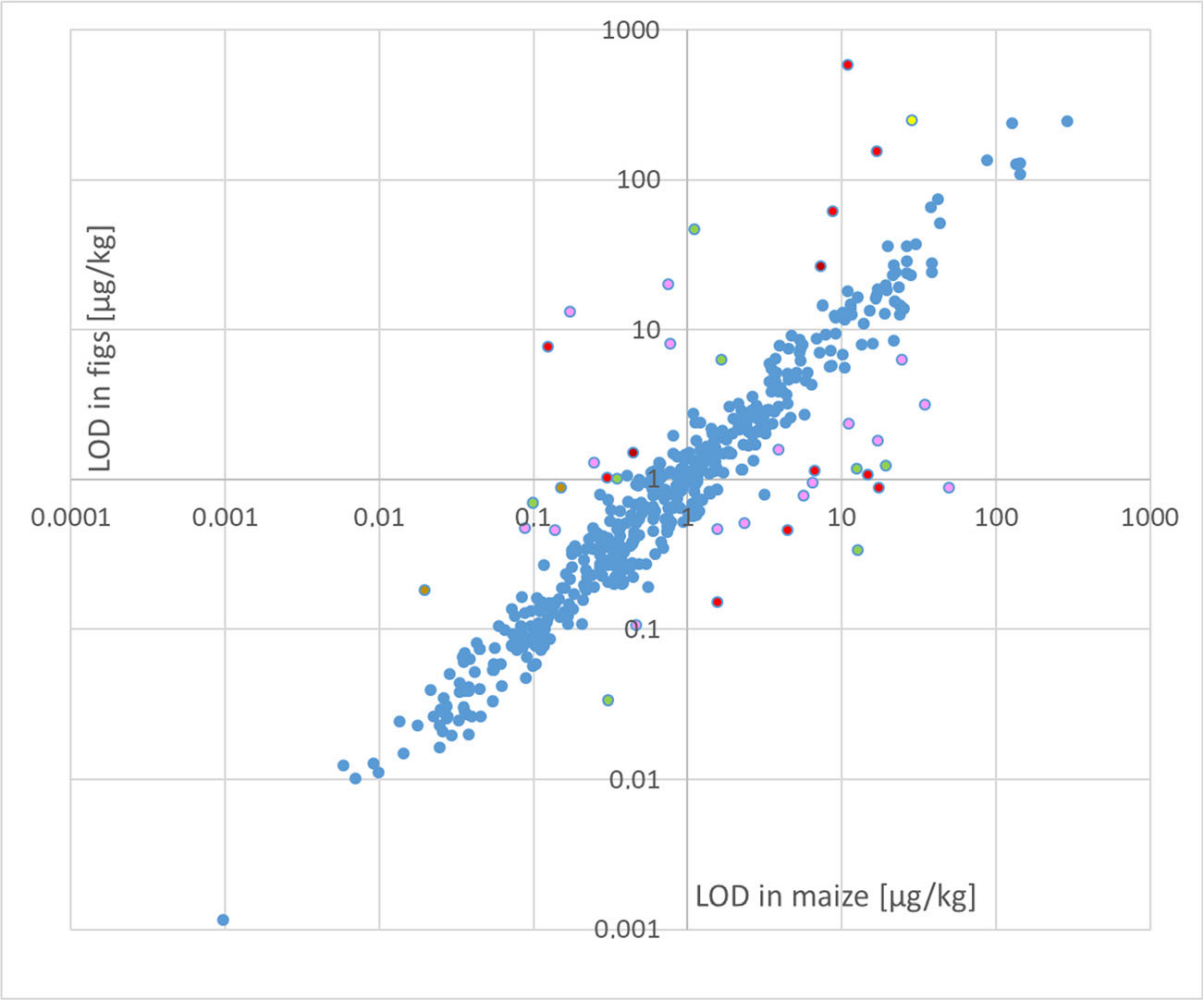
Comparison of LODs obtained for figs and maize. Each dot represents one analyte. Pink dots: interference or high baseline in one matrix; red dots: low recovery of extraction in one matrix; green dots: no true blank available for one matrix, thus hampering spiking at lower levels; brown dots: High lot-to-lot variation in maize; yellow dots: instable compound

Numerical values for LOQs and LODs for all analytes in maize and figs are given in ESM Table S8. For 89.6% of the investigated analytes, the difference of the numerical values obtained for figs and maize was less than a factor of 2 (see Fig. 7 and ESM Table S9 for numerical values). For the remaining analytes, the related discrepancies are caused by the non-availability of true blanks (thus hampering data evaluation of a lower spiking level) or an interference/increased baseline for one of the two matrices, or by differences in recoveries of the extraction step (Fig. 7). In view of this, the re-determination of LOD/LOQ for each new matrix does not seem to be essential unless the analysis of samples spiked at a high level (as proposed above) reveals the presence of interferences/noise for particular analytes.

The EURACHEM guide is more convenient for estimating LOQ/LOD for multi-analyte methods compared with other approaches requiring equidistant calibration levels close to the LOD [1], which is a huge effort in case of several hundred analytes. The EURACHEM approach uses a multiplication factor of 10 for s_0_ (corresponding to an RSD of 10%), whereas most other guidelines state RSD ≤ 20% as the related criterion. Furthermore, it is not regulated that the data used for determining the sensitivity have to be derived from different individual samples, thus neglecting the contribution of relative matrix effects to the precision. As a result, the numerical values determined for LOQ and LOD in this study are a very conservative estimation. Indeed, a large part of the PT results submitted for aflatoxins and ochratoxin A in baby food exhibited a satisfactory *z*-score, although these were close to or even below the estimated LOQs. A recent study has shown that the LODs depend on the condition of the LC-MS instrument and on the method used for estimation as well and concluded that *the LOD* of an LC-MS-based method does not exist [26]. For this reason, we believe that for a method with a very broad scope, it is most pragmatic to estimate the LOD/LOQ using a fast, but conservative approach and to apply the related values for all matrices. The latest generation of LC-MS instruments is sufficiently sensitive to guarantee that for most analytes, any concentration below the related levels are irrelevant anyway, exceptions being aflatoxins and ochratoxin A for the present method or banned substances in the field of residue analysis of pesticides and veterinary drugs.

### Critical assessment of the employed dilute-and-shoot method

In LC-MS-based multi-analyte determination covering dozens or even hundreds of analytes, the employment of clean-up steps optimized for each metabolite is not an option due to the wide range of physicochemical properties/polarities, which is true even for the comparatively small set of mycotoxins addressed by regulations. As a result, most of the related methods rely on stable isotope-labelled internal standards after a crude QuEChERS-based salting-out step (sometimes in combination with dispersive solid phase extraction) to compensate absolute and relative matrix effects, which have been confirmed to be a main contributor to the method uncertainty in this study. As the application of this internal standardization was considered to be out of the focus of this work (but could be easily implemented in principle, e.g., by adding the internal standards via an autosampler program), the method presented herein is not intended for measurements of mycotoxins addressed by regulatory limits on the highest metrological level (e.g., inter-laboratory certification studies of reference materials) or to be used as official reference method. Nevertheless, we consider the method performance to be much better than what is expected for a mere screening method (which seems to be still regarded as the main purpose of LC-MS/MS-based multi-analyte methods in the literature) in view of the rate of > 94% of satisfactory *z*-scores indicating its competitiveness with the methods of the other proficiency test participants (that partially include stable isotopically labelled standards).

This indicates that in the case of the latest generation of mass spectrometers, the obvious advantages of an LC-MS/MS-based “dilute and shoot” method, i.e., its multi-analyte capability, simplicity, and speed, are not outweighed by a highly compromised performance due to the presence of co-eluting matrix constituents. Indeed, we have neither observed an accelerated contamination of the mass spectrometer (we have found two preventive maintenances per year to be sufficient) nor a decreased accuracy/precision for analyte/matrix combinations exhibiting comparatively strong absolute matrix effects. As other authors preferred to omit a clean-up step such as dispersive solid phase extraction as well [27], we expect that the importance of this approach (in combination with internal standardization where applicable) will further increase. Therefore, the need for specification/guidance as outlined in this paper becomes more and more pressing.

## Conclusions

Current guidelines for method validation are of limited value for LC-MS-based methods with a broad scope of analytes, as there seems to be a severe imbalance between the relevance of the various performance parameters and the related workload foreseen for their determination. In particular, matrix effects are neglected in several ways: First of all, there are contradicting opinions whether recovery or apparent recovery has to meet specific target ranges, which is much more difficult to achieve in the latter case at least in our method (53–83% vs. 84–94%). The use of matrix matching or stable isotopically labelled standards (the latter being hardly applicable for hundreds of analytes though) renders this point hypothetical, but there is a lack of guidance whether or not a method including compensation of absolute matrix effects is considered to exhibit an acceptable performance if the signal is tremendously suppressed (e.g., by more than a factor of 10).

The need for guidance is even more pressing for relative matrix effects, particularly as they cannot be compensated by matrix matching. One might deduce that the precision of spiked replicates derived from different samples should still comply with the related criteria (e.g., RSD_r_ ≤ 20%), despite the fact that the definitions given for “replicates” in validation guidelines rather suggest the use of technical replicates from a single sample for the determination of repeatability and reproducibility. Indeed most analyte/matrix combinations investigated in this work were compliant in terms of repeatability. However, the comparison of data obtained for RSD_r_ and on RSD_WLR_ in seven individual samples and seven technical replicates of the same sample, respectively, indicates that priority should be given to the former upon method transfer in order to estimate the contribution of relative matrix effects to the uncertainty of the method in the new matrix.

The option of reducing the workload by validating just one matrix per commodity group is not recommended in view of the statistically significant differences between apparent recoveries in the matrices from the same matrix group as shown in Table 3. Instead, at least a minimum effort to characterize apparent recoveries as suggested below should be made upon transfer of the method to a new matrix. In contrast to that, the workload involved in the investigations of low analyte concentrations (implying time-consuming manual inspection of all related chromatograms) might be substantially decreased. Matrix effects, extraction efficiencies, and apparent recoveries were found to be independent from the analyte concentrations and the LODs/LOQs determined in maize and figs showed a reasonable agreement, suggesting that establishing a matrix-independent conservative estimation is feasible.

Concluding, for transfer of an existing multi-method covering hundreds of analytes to a new matrix, we propose the analysis of replicates obtained from seven individual samples spiked at a high concentration level under repeatability conditions as a compromise for obtaining maximal information of method performance with a reasonable effort. This minimizes time-consuming manual inspection of chromatograms, determines apparent recovery, includes relative matrix effects as the main contributor to the uncertainty of the method, and reveals if there is any interference/elevated baseline noise that might pose the need to adapt a previously established general LOD/LOQ of a given analyte to this particular matrix.

## Electronic supplementary material

ESM 1 (PDF 370 kb)

ESM 2 (XLSX 50 kb)

ESM 3 (XLSX 72 kb)

ESM 4 (XLSX 588 kb)

ESM 5 (XLSX 44 kb)

ESM 6 (XLSX 303 kb)

ESM 7 (XLSX 96 kb)

ESM 8 (XLSX 144 kb)

ESM 9 (XLSX 144 kb)

ESM 10 (XLSX 52 kb)
