# Supplementary material for: Validation of an LC-MS/MS-based dilute-and-shoot approach for the quantification of > 500 mycotoxins and other secondary metabolites in food crops: challenges and solutions
Source: Anal Bioanal Chem. 2020 Feb 20;412(11):2607–20. doi: 10.1007/s00216-020-02489-9 (PMC7136310; doi:10.1007/s00216-020-02489-9)
Supplement: Supplementary file 1 — (PDF 370 kb) [file 216_2020_2489_MOESM1_ESM.pdf]

Analytical and Bioanalytical Chemistry

Electronic Supplementary Material

Validation of an LC-MS/MS based dilute-and-shoot approach for the quantification of >500 mycotoxins and other secondary metabolites in food crops: challenges and solutions

Michael Sulyok, David Stadler, David Steiner, Rudolf Krska

Additional files available under 10.1007/s00216-020-02489-9

Fig. S1 Correlation of recoveries of extraction with retention time

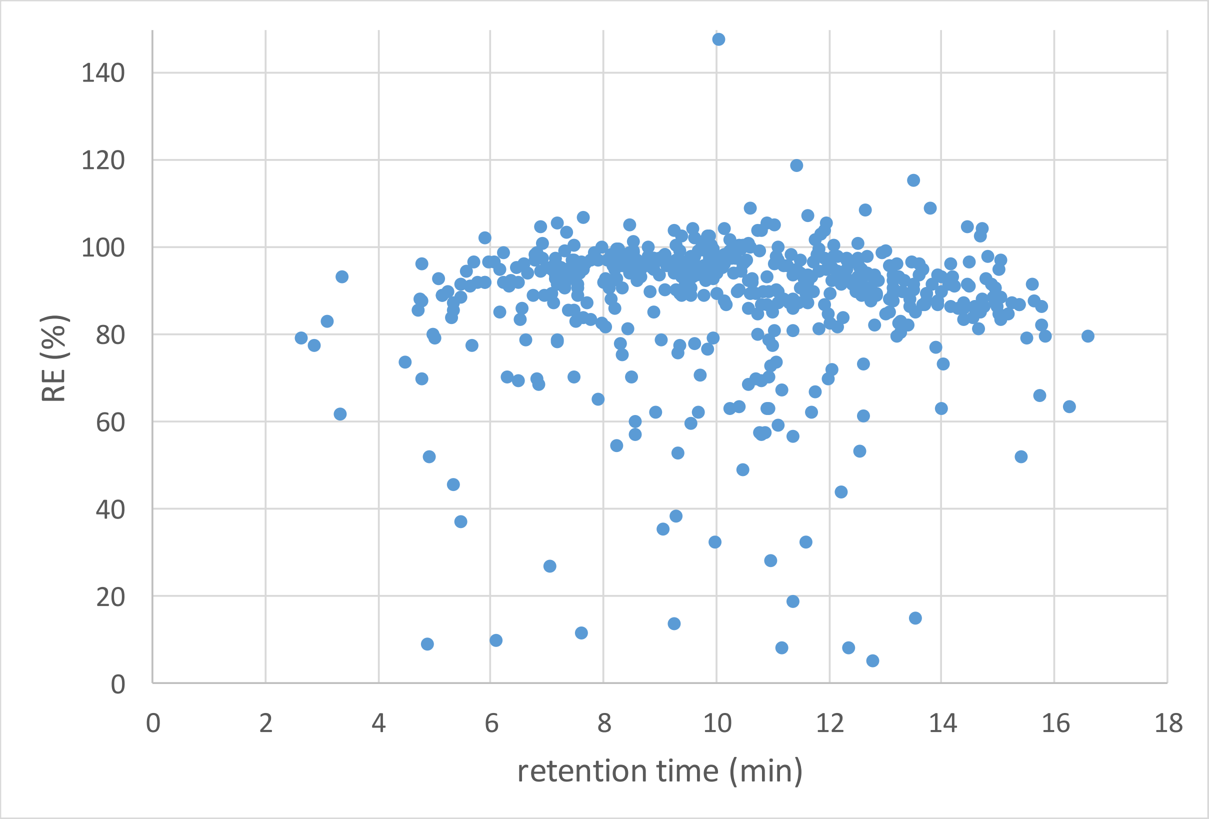

Fig. S1a Correlation of recoveries of extraction obtained in maize with retention time

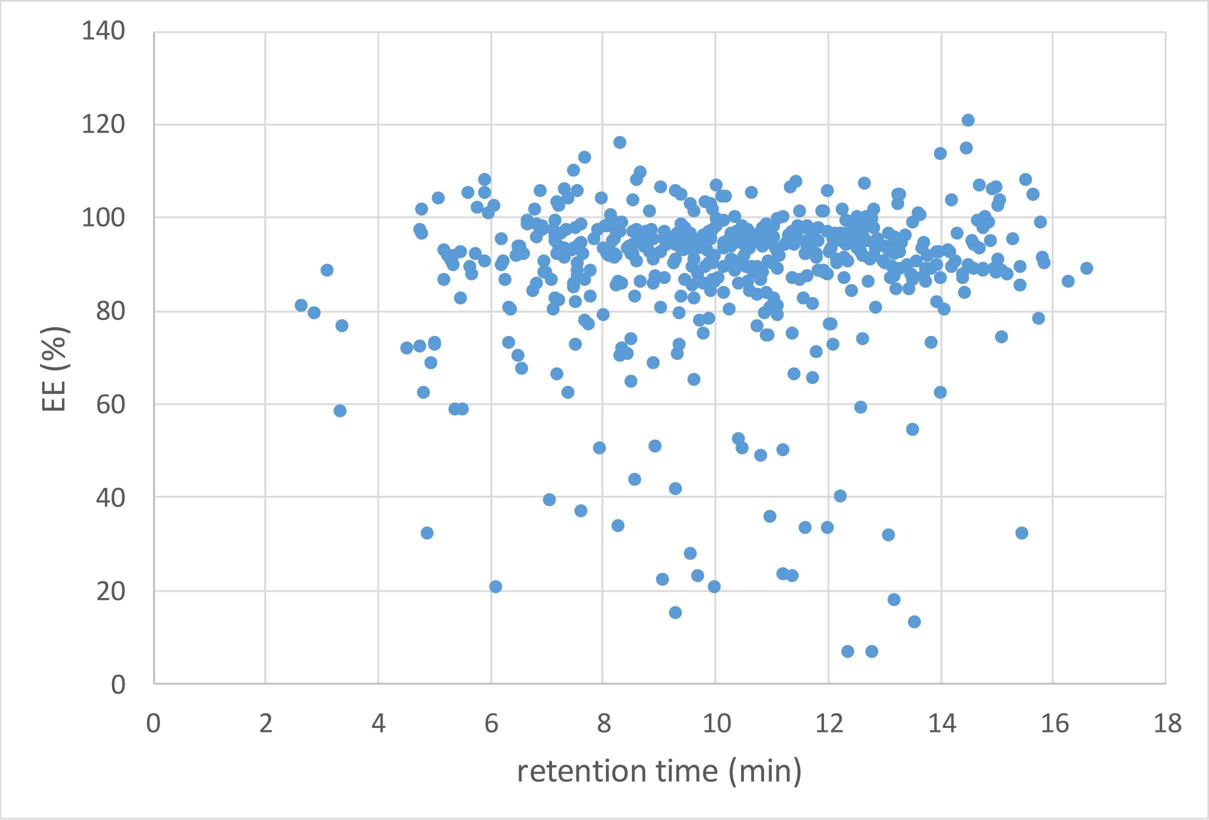

Fig. S1b Correlation of recoveries of extraction obtained in wheat with retention time

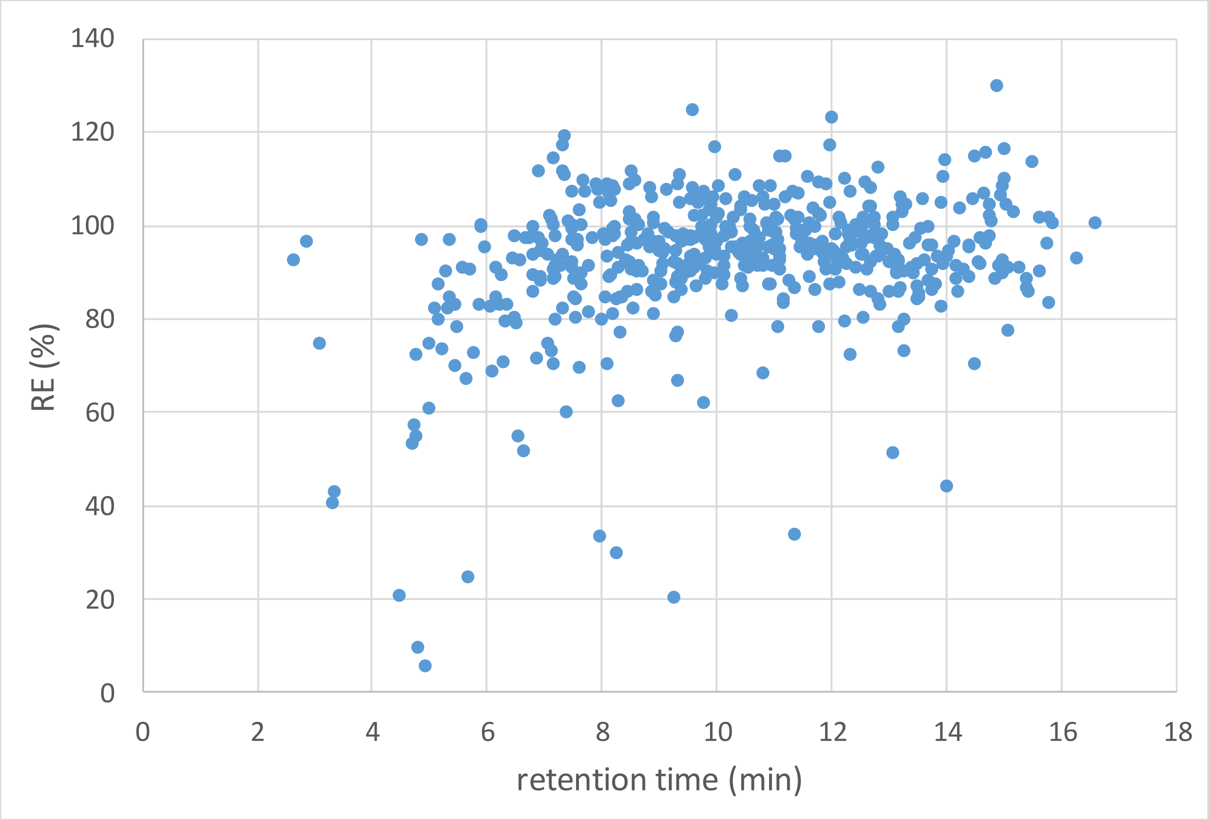

Fig. S1c Correlation of recoveries of extraction obtained in grapes with retention time

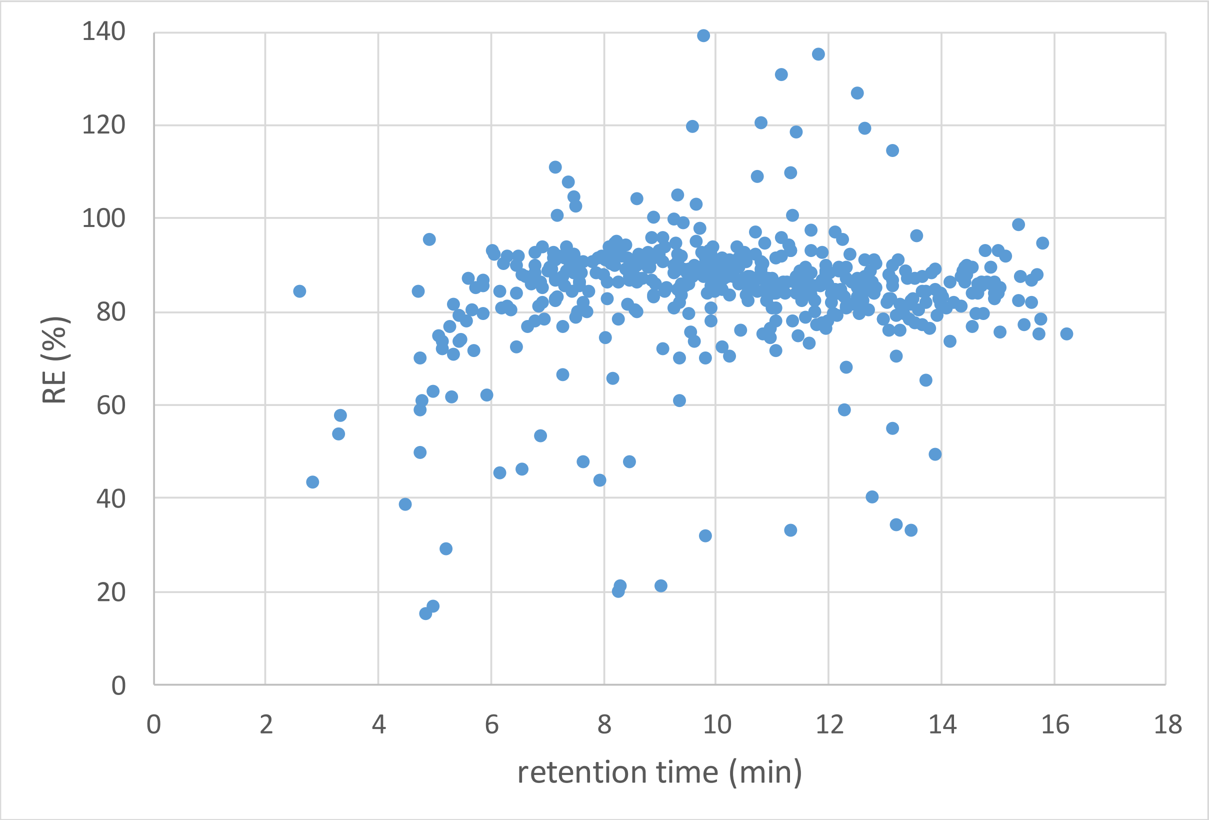

Fig. S1d Correlation of recoveries of extraction obtained in figs with retention time

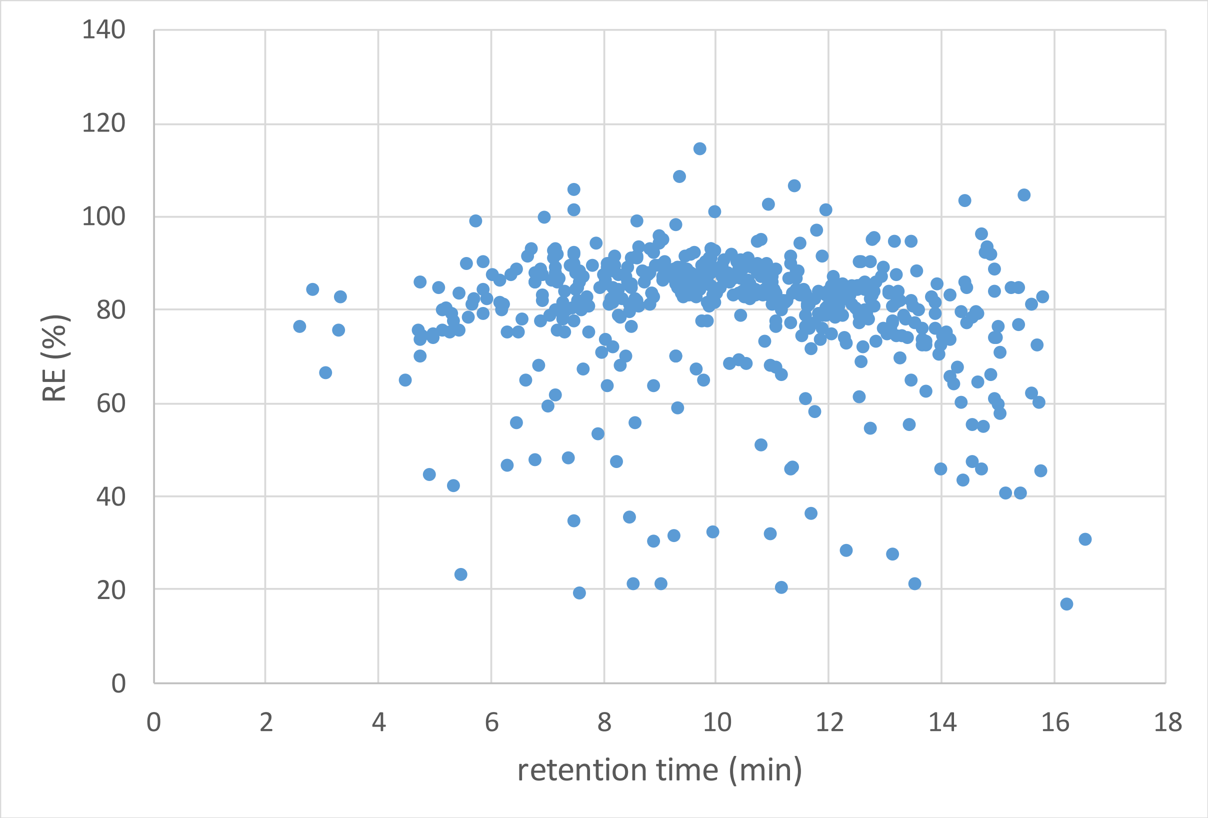

Fig. S1e Correlation of recoveries of extraction obtained in walnuts with retention time

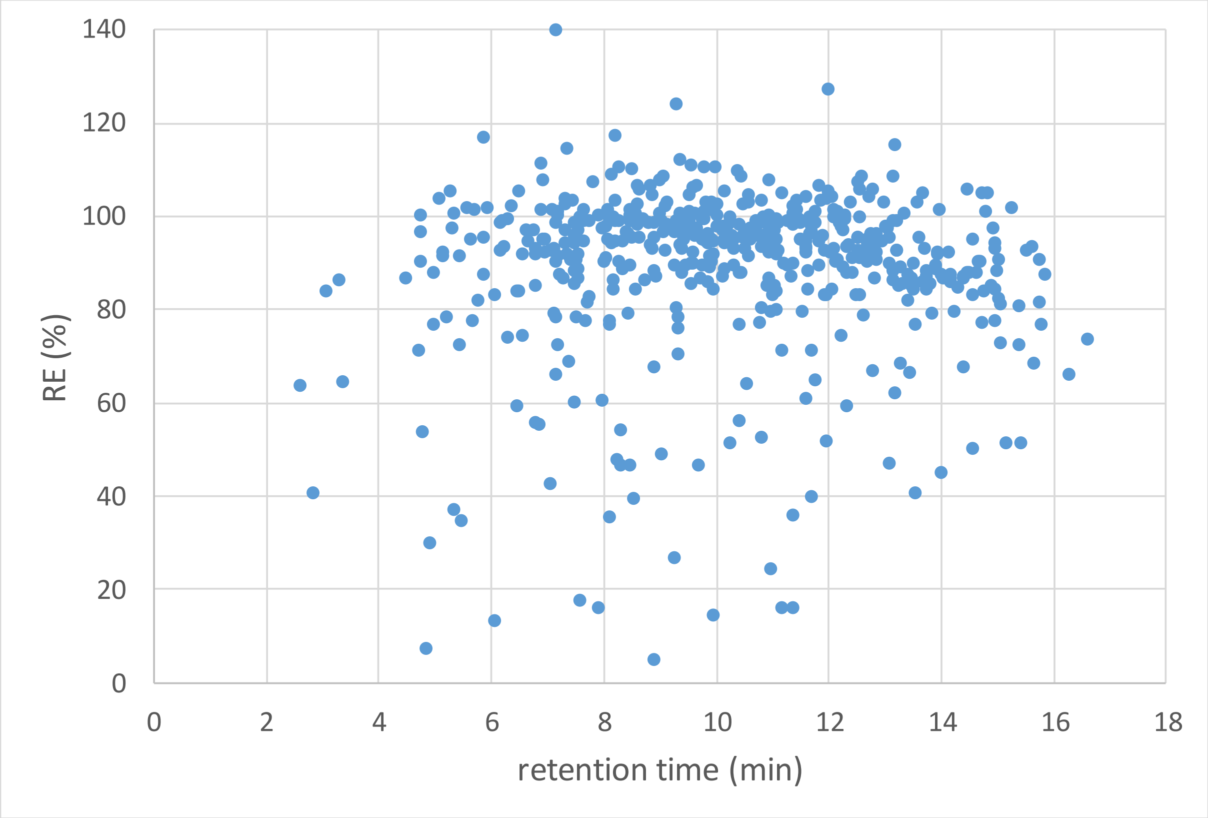

Fig. S1f Correlation of recoveries of extraction obtained in almonds with retention time
